# Synthesis, crystal and electronic structure of BaLi_*x*_Cd_13–*x*_ (*x* ≈ 2)

**DOI:** 10.3389/fchem.2022.991625

**Published:** 2022-09-07

**Authors:** Wanyue Peng, Sviatoslav Baranets, Svilen Bobev

**Affiliations:** Department of Chemistry and Biochemistry, University of Delaware, Newark, DE, United States

**Keywords:** Cd, Li, single-crystal X-ray diffraction, crystal structure, synthesis

## Abstract

A new ternary phase has been synthesized and structurally characterized. BaLi_*x*_Cd_13–*x*_ (*x* ≈ 2) adopts the cubic NaZn_13_ structure type (space group *Fm* $\bar{3}$ *c*, Pearson symbol *cF*112) with unit cell parameter *a* = 13.5548 (10) Å. Structure refinements from single-crystal X-ray diffraction data demonstrate that the Li atoms are exclusively found at the centers of the Cd_12_-icosahedra. Since a cubic BaCd_13_ phase does not exist, and the tetragonal BaCd_11_ is the most Cd-rich phase in the Ba–Cd system, BaLi_*x*_Cd_13–*x*_ (*x* ≈ 2) has to be considered as a true ternary compound. As opposed to the typical electron count of ca. 27*e*-per formula unit for many known compounds with the NaZn_13_ structure type, BaLi_*x*_Cd_13–*x*_ (*x* ≈ 2) only has ca. 26*e*-, suggesting that both electronic and geometric factors are at play. Finally, the bonding characteristics of the cubic BaLi_*x*_Cd_13–*x*_ (*x* ≈ 2) and tetragonal BaCd_11_ are investigated using the TB-LMTO-ASA method, showing metallic-like behavior.

## 1 Introduction

In recent papers, we described new results from our exploratory work in the Ba–Li–In–Ge (Ovchinnikov and Bobev, 2019) and Ba–Li–Cd–Ge systems (Baranets et al., 2021b). As noted therein, our synthetic approach employed molten In and Cd as metal fluxes, allowing us to grow crystals of the quaternary phases, BaLi_2+*x*_In_2–*x*_Ge_2_ (0 ≤ *x* ≤ 0.66) and BaLi_2_Cd_2_Ge_2_ (CaCu_4_P_2_ type, space group *R* $\bar{3}$ *m*; Pearson code *hR*7). The structure of this indium-germanide was found to respond to small changes in the chemical composition (alterations of the Li/In ratio) by virtue of cleaving homoatomic In–In bonding (Ovchinnikov and Bobev, 2019). BaLi_2_Cd_2_Ge_2_ (Baranets et al., 2021), on the other hand, was found to be less flexible as far as the structure is concerned, just like its quaternary BaMg_2_Li_2_Ge_2_ analog (Zürcher et al., 2001). On this note, it is instructive to mention that despite the resemblance between the chemical compositions, and structures of BaMg_2_Li_2_Ge_2_ and BaLi_2_Cd_2_Ge_2_ as compared to BaLi_2+*x*_In_2–*x*_Ge_2_, all three phases show subtly different bonding characteristics. The differences are most pronounced between the structures with trivalent In vs. those with divalent Mg and Cd (Zürcher et al., 2001; Ovchinnikov and Bobev, 2019; Baranets et al., 2021b), attesting to the importance of the valence electron count for the peculiarities of each structure.

Cadmium metal is known to have considerable toxicity with a destructive impact on most living systems. Due to its low boiling point, Cd is also far from an ideal solvent for high-temperature reactions. However, the crucial role of Cd in the flux growth of new intermetallic compounds makes the current study fit into this special issue on *Crystal Growth Under Extreme Conditions*. BaLi_2_Cd_2_Ge_2_ (Baranets et al., 2021) together with Yb_2_CdSb_2_ (Xia and Bobev, 2007a), Sr_2_CdAs_2_ (Wang et al., 2011), Ba_2_CdAs_2_ (Wang et al., 2011), Eu_2_CdAs_2_ (Wang et al., 2011), Ba_11_Cd_8_Bi_14_ (Xia and Bobev, 2006a), *RE*_2_CdGe_2_ (*RE* = Pr, Nd, Sm, Gd–Yb; Y) (Guo et al., 2012), Ba_2_Cd_3_Bi_4_ (Xia and Bobev, 2006b), *A*_14_Cd_1+*x*_ *Pn*_11_ (0 ≤ *x* ≤ 0.3; *A* = Sr, Eu; *Pn* = As, Sb) (Makongo et al., 2015), Sr_3_Cd_8_Ge_4_ (Suen et al., 2018), Eu_3_Cd_8_Ge_4_ (Suen et al., 2018), and Eu_10_Cd_6_Bi_12_ (Xia and Bobev, 2007b) from our laboratory have come into being by employing molten Cd as a flux for the synthesis. This is also the case with the title compound, BaLi_*x*_Cd_13−*x*_ (*x* ≈ 2). As mentioned above, BaLi_*x*_Cd_13−*x*_ (*x* ≈ 2) was serendipitously obtained during the exploratory work in the *AE*–Li–Cd–Si and *AE*–Li–Cd–Ge systems (*AE* = alkaline earth metals Ca, Sr, Ba, and the nominally divalent Eu, Yb). As a result of such work, the new cubic BaLi_*x*_Cd_13–*x*_ (*x* ≈ 2) phase was discovered. It is the first structurally characterized compound between the respective elements and crystallizes with the common NaZn_13_ structure type (space group *Fm* $\bar{3}$ *c*, Pearson symbol *cF*112). Yet, a binary phase BaCd_13_ with this structure is unknown, making BaLi_*x*_Cd_13–*x*_ (*x* ≈ 2) a true ternary compound.

The crystal growth from Cd flux, the structural characterization, as well as a brief analysis of the chemical bonding of BaLi_*x*_Cd_13–*x*_ (*x* ≈ 2) and BaCd_11_ are the main subject of discussion in this paper.

## 2 Materials and methods

### 2.1 Synthesis

The starting materials were purchased from Alfa Aesar, all with stated purity of 99.9 wt% or better. The metals were stored in an argon-filled glovebox and used as received. The surface of the Li rod was cleaned with a scalpel blade prior to cutting and using it.

Single crystals were synthesized *via* the flux growth method. As stated already, reactions aimed at *AE*Li_2_Cd_2_Ge_2_ (*AE* = alkaline-earth metal) were the starting point for this research, (Baranets et al., 2021b), and the title compound BaLi_*x*_Cd_13–*x*_ (*x* ≈ 2) was identified as a minor side product of such experiment. Subsequently, the samples were prepared without Ge in the elemental mixtures, where excessive stoichiometric amounts of Cd served as the flux for all compositions. A starting composition of Ba:Li:Cd: of 1:12:20 was used for BaLi_*x*_Cd_13−*x*_ (*x* ≈ 2). The 6-fold excess Li was chosen due to its high vapor pressure and losses at the reaction temperature. The corresponding amounts of elements (Ba rod, Cd shot, Zn shot, Ca shot, Yb shot, Li rod) were loaded into alumina crucibles. A piece of quartz wool was placed at the bottom of a fused silica ampoule. The alumina crucible was then loaded to the bottom of an ampoule. Another piece of quartz wool was placed on top of the crucible without touching the elements inside. The ampoules were sealed under a vacuum level of ca. 30 millitorr.

All the samples in this study were heated in muffle furnaces with a temperature profile as the following: 100°C → 20°C/h to 200°C → 50°C/h to 700°C → held at 700°C for 12.5 h → 4°C/h to 550°C. At this point of the crystal growth, the sealed tube was taken out from the furnace, flipped, and the molten metallic flux was separated from the grown crystals by centrifugation. After that, the sealed ampoule was brought in the glovebox and break-opened. Inspection of the specimen under an optical microscope revealed the presence of many small, grey crystals with a metallic luster, usually clustered together. Single-crystal X-ray diffraction confirmed them to be the new cubic BaLi_*x*_Cd_13−*x*_ (*x* ≈ 2) phase. A portion of the crystallites was then ground into fine powders with a mortar and pestle for powder X-ray diffraction, attesting the presence of the cubic phase in the bulk. The experiment was repeated in a Nb-tube to confirm that the results are repeatable in both alumina and Nb containers. Such reactions also verified that no inadvertent reduction of the Al_2_O_3_ can become a source of Al metal, as experienced recently by us in another materials system (Baranets and Bobev, 2021a).

Following the structure elucidation of BaLi_*x*_Cd_13–*x*_ (*x* ≈ 2), reactions aimed at *AE*Li_*x*_Cd_13–*x*_ (*x* ≈ 2) (*AE* = Ca, Sr, Eu, Yb) were set up with the same nominal compositions as the ones described in the preceding paragraph, but they were unsuccessful. An attempt to grow crystals from Cd-flux reaction but without Li in the nominal mixture resulted in the growth of the known binary phase BaCd_11_ (Sanderson and Baenziger, 1953). Since its structure has not been refined from single-crystal X-ray diffraction data and since the quality of the obtained crystals was excellent, herein we supply this information as well.

Crystals of BaLi_*x*_Cd_13−*x*_ (*x* ≈ 2) do not appear to degrade in air over a period of 1 week. Powder X-ray diffraction patterns also show the polycrystalline material to be air- and moisture-stable for the same amount of time and possibly even longer.

### 2.2 Powder X-ray diffraction and single-crystal X-ray diffraction

The X-ray powder diffraction patterns were collected using a Rigaku Miniflex powder diffractometer utilizing Ni-filtered Cu *K*_α_ radiation (*λ* = 1.5418 Å) and were used for phase identification only. All additional structural work was done using single-crystal X-ray diffraction methods.

Single-crystal X-ray diffraction measurements (SC-XRD) were performed using a Bruker APEX II diffractometer with monochromated Mo K*α* radiation. Single crystal geometries are typically in block shape, with each side smaller than 60 *µ*m in length. Crystals were selected under a microscope in dry Paratone-N oil. The measurements were conducted at a temperature of 200 K. Data integration and semiempirical absorption correction were performed with the Bruker-supplied software (Bruker AXS Inc, 2014). The crystal structure was solved with the intrinsic phasing method and was refined with full-matrix least-squares minimization on *F* ^2^ using ShelXL (Sheldrick, 2015). Olex2 software was used as a graphical interface (Dolomanov et al., 2009). Atomic coordinates of all compounds reported in this paper were standardized with the Structure Tidy program (Gelato and Parthé, 1987). All sites were refined with anisotropic displacement parameters. Final difference Fourier map was flat and featureless. Selected crystallographic data are summarized in Table 1.

**TABLE 1:** Selected crystallographic data and structure refinement parameters for BaLi_*x*_Cd_13–*x*_ (*x* ≈ 2).

Formula	BaLi_2.14(4)_Cd_10.86_
Formula Weight/g mol^−1^	1,373.0
Radiation, *λ*	Mo *K*_α_, 0.71073 Å
Temperature/K	200 (2)
Crystal system	Cubic
Space Group	*Fm* $\bar{3}$ *c* (no. 226)
*Z*	8
*a/*Å	13.5548 (10)
*V*/Å^3^	2,490.5 (6)
*ρ*_*calc*_ */*g cm^−3^	7.32
*µ*_MoK*α*_/cm^−1^	211.7
Reflections: parameters	187: 11
*R*_1_ (*I* >2σ_(*I*)_)^a^	0.0156
*R*_1_ (all data)^a^	0.0165
*wR*_2_ (*I* >2σ_(*I*)_)^a^	0.0261
*wR*_2_ (all data)^a^	0.0262
Largest peak; deepest hole/*e* Å^−3^	0.45; –0.73^b^

a $R_{1}=\Sigma \left\|F_{o}|-|F_{c}\right\|/\Sigma \left|F_{o}\right|$ ; $wR_{2}={\left[\Sigma \left[w{\left(F_{o}^{2}-F_{c}^{2}\right)}^{2}\right]\right]/\left[\Sigma \left[w{\left(F_{o}^{2}\right)}^{2}\right]\right]}^{1/2}$ , where $w=1/\left[\sigma^{2}F_{o}^{2}+{\left(\text{0}\text{.}0032P\right)}^{2}+33.84\right]$ , and $P=\left(F_{o}^{2}+2F_{c}^{2}\right)/3$ ;

b The largest peak and the deepest hole are 1.9 Å away from Cd/Li and 0.8 Å away from Cd/Li, respectively.

One specific aspect of the refinements concerning the site occupation factors (SOF) requires a special mention. All SOFs were checked by freeing an individual SOF, while other variables were kept fixed. No statistically significant deviations were observed for the SOF of the Ba site (8*a*); the Li position (8*b*) indicated a freely-refined SOF of ca. 105%. The detected “over-occupation” of the Li atom barely had statistical significance (deviations were within ca. 3-4σ). The Cd site (96*i*) showed approximately 94–95% occupancy (within ca. 8-9σ), which is indicative of the existence of either 1) vacancies, or 2) potential disordering with a lighter element, such as Li in this case. The model with Li/Cd co-occupation was chosen on the basis of expected greater electronic structure stability of BaLi_*x*_Cd_13–*x*_ (*x* ≈ 2) vs. BaLiCd_12–*x*_ (*x* ≈ 0.6–0.7). We must also note that ignoring the above-mentioned signs for a small disorder on the Cd site (96*i*) and refining an ordered BaLiCd_12_ model leads to a converging least-squares minimization, however, with increased R-values (*R*_1_ = 0.0182; *wR*_2_ = 0.0443) compared to those in presented Table 1 for the BaLi_*x*_Cd_13–*x*_ (*x* ≈ 2) structure. Residual difference peak and hole in the Fourier synthesis map were also much higher, therefore, this possibility was excluded from further consideration. Further details of the structural work are discussed later on. The corresponding crystallographic information files (CIF) for BaLi_*x*_Cd_13–*x*_ (*x* ≈ 2) and BaCd_11_ have been deposited with CSD, and the data for this paper can be obtained free of charge *via* http://www.ccdc.cam.ac.uk/conts/retrieving.html (or from the CCDC, 12 Union Road, Cambridge CB2 1EZ, United Kingdom; Fax: +44-1223-336033; E-mail: deposit@ccdc.cam.ac.uk). Depository numbers are 2184513 (BaLi_*x*_Cd_13–*x*_) and 2184514 (BaCd_11_).

### 2.3 Electronic structure calculations

To investigate the chemical bonding of all compositions, electronic structure calculations were performed within the local density approximation (density functional theory) using the TB-LMTO-ASA program (Tank et al., 1994). Experimental unit cell parameters and atomic coordinates obtained in this study were used as the input parameters in our calculation. In order to satisfy the atomic sphere approximation (ASA), we employed von-Barth-Hedin functional (Von Barth and Hedin, 1972) and introduced empty spheres during the calculation. The Brillouin zone was sampled by 1,000 *k*-point grid. Electronic density of states (DOS), atom-projected electronic density of states (PDOS), and crystal orbital Hamilton population (COHP) were calculated with modules in the LMTO program (Steinberg and Dronskowski, 2018).

## 3 Results and discussion

### 3.1 Crystal structure

The structures of some *AM*_13_ compounds (space group *Fm* $\bar{3}$ *c*; *A* = K, Rb, Ca, Sr, Ba and *M* = Zn, Cd) were first reported by Ketelaar in 1937 (Ketelaar, 1937). The first material synthesized and characterized in this group was NaZn_13_, which has become the archetype of the family (Pearson symbol *cF*112) (Villars and Calvert, 1991). The structure is relatively complex, although there are only three unique positions in the asymmetric unit. For the herein discussed BaLi_*x*_Cd_13–*x*_ (*x* ≈ 2), they are listed in Table 2. The overall NaZn_13_ structure is often referred to as cage-like and can be visualized as a face-centered cubic arrangement of snub cubes (Zn1, at site 96*i*) surrounding the Na atoms (at site 8*a*). The third position is taken by a Zn atom (Zn2, at site 8*a*), centering a Zn_12_-icosahedron (notice the atypical 12-fold Zn2 coordination by Zn1). A more detailed structural description can be found in Ref (Nordell and Miller, 1999). and Ref. (Häussermann et al., 1998).

**TABLE 2:** Atomic coordinates and equivalent displacement parameters (Å^2^) for BaLi_*x*_Cd_13–*x*_ (*x* ≈ 2).

Atom	Wyckoff symbol	Site symmetry	*x*	*y*	*z*	*U*_*eq*_ ^a^
Ba	8*a*	4 3 2	1/4	1/4	1/4	0.0142 (3)
Cd/Li^b^	96*i*	*m . .*	0	0.11998 (3)	0.17782 (3)	0.0154 (2)
Li	8*b*	*m* $\bar{3}$	0	0	0	0.018 (6)

a *U*_eq_ is defined as one third of the trace of the orthogonalized *U*_ij_ tensor.

b Refined as mixed-occupied Cd/Li in a ratio of 0.905 (4)/0.095.

There are over a hundred compounds reported to crystallize in the NaZn_13_ structure type (Villars and Calvert, 1991). In the past 2 decades, the NaZn_13_ family has been expanded to include many ternary counterparts, some of which are cubic alloys, such as EuCu_*x*_Al_13−*x*_ (Phelan et al., 2012) and *RE*Co_13−*x*_Ga_*x*_ (*RE* = La, Ce, Pr, Nd, and mischmetal) (Weitzer et al., 1990), while some other show crystallographic ordering. Examples of the latter group are BaLi_7_Al_6_ (Häussermann et al., 1998), *AM*_*x*_ *T*_13−*x*_ (*A* = Ba, Sr, La, Eu, *M* = Cu and Ag; *T* = Al, Ga and In; *x* = 5–6.5.) (Nordell and Miller, 1999), *A*Cu_9_ *Tt*_4_ (*A* = Ca, Sr, Ba, Eu; *Tt* = Si, Ge, Sn) (Schäfer et al., 2012), and BaAu_*x*_Zn_13−*x*_ (Gupta and Corbett, 2012). One should notice that most structurally characterized phases are known to follow the “1-13” stoichiometry, while other members are shown to be slightly off-stoichiometric, such as SrZn_13–*x*_ (Wendorff and Röhr, 2006), and EuZn_13–*x*_ (Saparov and Bobev, 2008), where the Zn-deficiency is necessitated for the sake of attaining more favorable valence electron count. More discussion on that topic will follow later. In some cases, particularly among the ternaries, ordering of the elements leads to cubic to tetragonal distortion (*Fm* $\bar{3}$ *c* to *I*4/*mcm*) as seen on the examples of *A*Cu_9_ *Tt*_4_ (*A* = Ca, Sr, Ba, Eu; *Tt* = Si, Ge, Sn) (Schäfer et al., 2012), but this is not the case here; the symmetry of BaLi_*x*_Cd_13–*x*_ (*x* ≈ 2) is strictly cubic.

**FIGURE 1:**
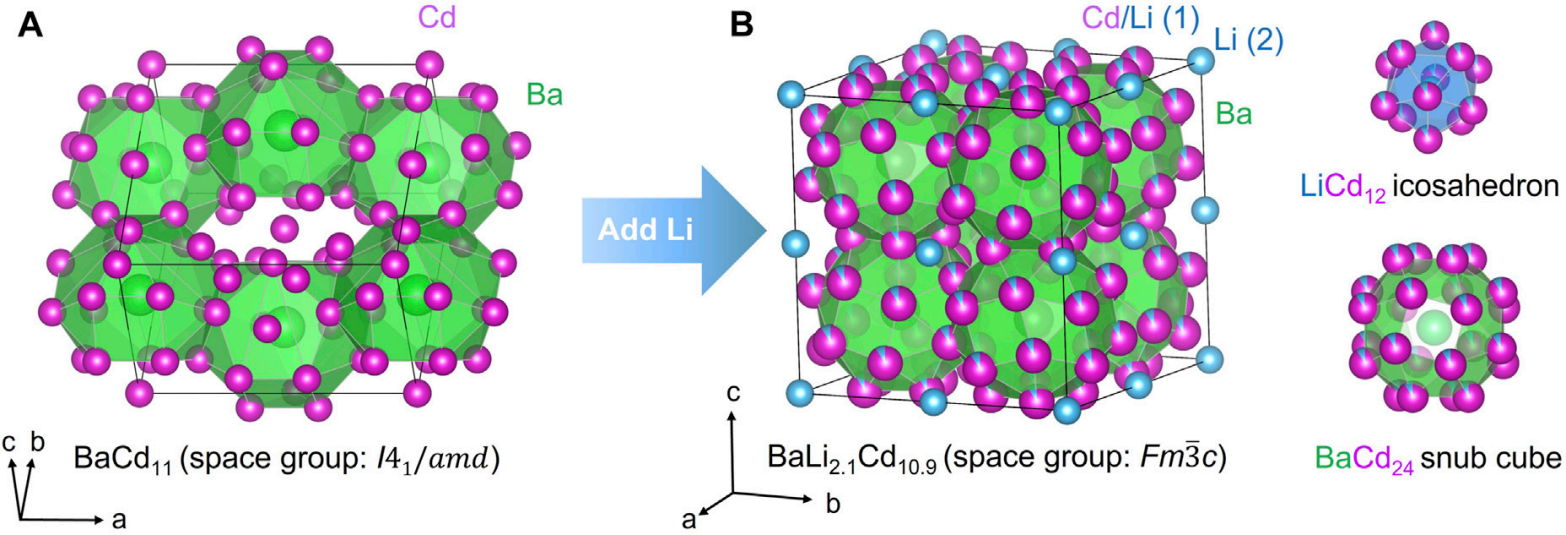
**(A)** BaCd_11_ crystallizes in a tetragonal structure type. It cannot transition to the cubic NaZn_13_ structure even with the presence of excessive Cd. **(B)** BaLi_2*.*1_Cd_10*.*9_ crystallizes in the cubic NaZn_13_ structure type).

We also need to draw attention to the fact that while BaZn_13_ (rather BaZn_∼12.8_) is known to crystallize with the cubic NaZn_13_ structure type (Wendorff and Röhr, 2006), a binary compound BaCd_13_ with the said structure does not exist. The most Cd-rich phase is BaCd_11_ and it crystallizes in its own structure type (tetragonal space group *I*4_1_/*amd* (no. 141); Pearson symbol *tI*48) (Sanderson and Baenziger, 1953). Despite the compositional difference and despite crystallizing in different space groups, the NaZn_13_ and BaCd_11_ structure types share a lot in common in terms of the large polyhedra surrounding the Na and Ba atoms, respectively (Figure 1). In the tetragonal structure, each Ba atom is surrounded by 22 Cd atoms (Cd1), while the Cd2 atoms have 12 nearest neighboring Cd ones. A more comprehensive structural description of the BaCd_11_ structure type can be found in Ref. (Sanderson and Baenziger, 1953). Besides BaCd_11_, YbZn_11_ (Kuzma et al., 1965), SrCd_11_ (Köster and Meixner, 1965), CeZn_11_ (Zelinska et al., 2004), and PrZn_11_ (Iandelli and Palenzona, 1967) are also reported to form with the same structure type.

Apparently, the NaZn_13_ structure can only be stabilized with Ba and Cd only when a small amount of lithium is present, with BaLi_2.1_Cd_10.9_ being the final refined composition (Table 1). One lithium atom takes over the Zn2 site in the NaZn_13_ prototype structure, while the balance of lithium atoms per formula unit is admixed with Cd at the Zn2 site in a ratio of ca. 1:10 (Table 2). The consequence of the Li atoms being in such positions is that, instead of having the same kind of atoms on both the vertices and the centers of the icosahedra, the center of the 12-membered polyhedron is Li, while the vertices are mostly Cd atoms. We confirmed that the hypothetic cubic BaCd_13_ phase was inaccessible without Li (*vide supra*). In such a Cd-rich environment, the crystals that grow are the tetragonal BaCd_11_. The exact opposite scenario is observed when one moves up in group 12 to Zn—the most Zn-rich phase in the Ba–Zn binary system is the cubic BaZn_13_ (Wendorff and Röhr, 2006)) while tetragonal BaZn_11_ does not exist.

Based on the above and given the similarities between the two structure types, one may ask the question as to what is the role Li atoms play to stabilize the cubic BaLi_*x*_Cd_13–*x*_ (*x* ≈ 2) phase, and whether or not the homogeneity range can be expanded. Our experimental findings are limited to compositions close to the refined, and an unambiguous answer to the two questions is not possible at present. However, we can speculate that since most known compounds within the NaZn_13_ structural family show valence electron counts close to 27*e* ^−^ per formula unit (Häussermann et al., 1998; Nordell and Miller, 1999), one should expect, based on electronic arguments alone, that the composition BaLi_*x*_Cd_13–*x*_ (*x* ≈ 1) would be preferred. Recognizing that there are geometric factors at play, too, and that they might be in competition with the electronic factors, could lead to different conclusions. In fact, there has been much debate about the favorable conditions of which structure type (NaZn_13_ or BaCd_11_) is optimal, yet, no universal conclusion has been reached. The hard-sphere colloidal model shows that the ideal size ratio between *A* and *M* is between 0.49 and 0.63 (Hachisu and Yoshimura, 1980; Yoshimura and Hachisu, 1983; Bartlett et al., 1992; Shevchenko et al., 2005). Later, Hudson (Hudson, 2010) approached this problem with the optimal packing fraction analysis. It is shown that the majority of the *AM*_13_ compounds yield a close packing fraction between 0.69 and 0.77. However, multiple known compounds break this rule. A typical example is SrBe_13_ (Matyushenko et al., 1964) yielding a size ratio of 2, which is far from the ideal range mentioned previously. It can be also suggested, again from a purely geometrical perspective, that the size of the atom in the icosahedral environment (recall that the covalent radii of Li and Zn are nearly identical while Li and Cd differ (Pauling, 1960)) is also a contributing factor, and could tip the scales when it comes to the phase-preference between cubic NaZn_13_ vs. tetragonal BaCd_11_.

Therefore, we can argue that the composition BaLi_*x*_Cd_13–*x*_ (*x* ≈ 2) may be the limiting/preferred one for the stabilization of the NaZn_13_-type structure for *both* electronic and geometric reasons.

### 3.2 Electronic structure

The bonding characteristics of BaLi_2*.*1_Cd_10*.*9_ and BaCd_11_ are investigated *via* electronic structure calculations using the LMTO program (Tank et al., 1994). Accurate modeling of the electronic properties of BaLi_2*.*1_Cd_10*.*9_ with the LMTO program is possible, yet challenging, due to the mixed occupancy of Li and Cd on the 96*i* site. The input of the mixed occupancy to the model would only be possible by reducing the cubic symmetry to triclinic *P*1, while assigning corresponding positions to Li and Cd atoms according to the refined occupancy. To simplify the calculation and to avoid symmetry reduction, we evaluated the electronic properties of BaLi_2*.*1_Cd_10*.*9_ in its original symmetry without considering the existence of Li on the Cd site. The model employed was the idealized BaLiCd_12_ compound with an ordered cubic structure, where each Ba, Cd, and Li atom resides on an independent site. Atomic coordinates were taken from Table 2.

**FIGURE 2:**
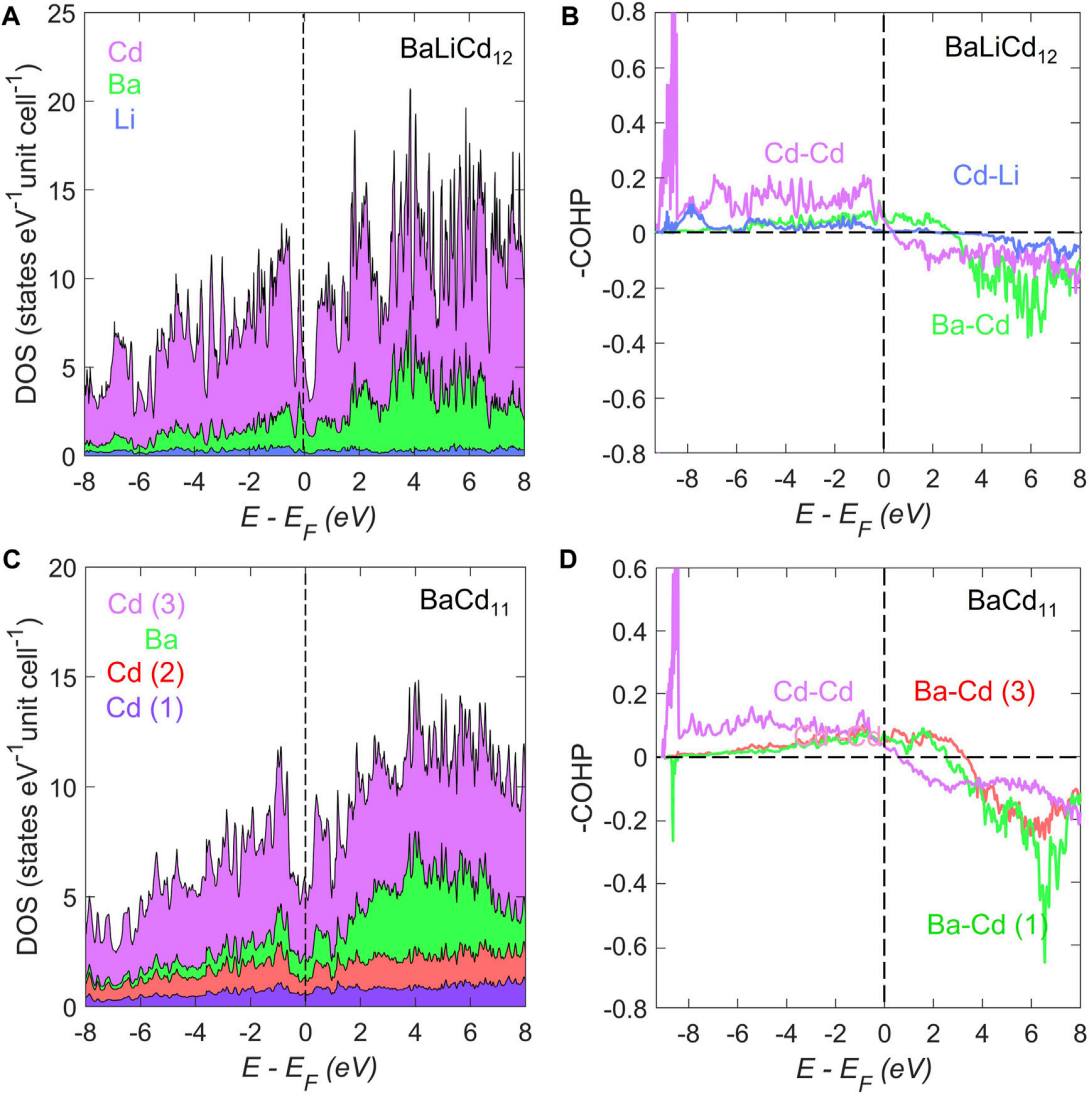
The atom-projected electronic density of states (DOS) for **(A)** BaLiCd_12_ and **(C)** BaCd_11_. The DOS of both compounds exhibit characteristics of metallic bonding. A relatively larger dip can be observed in the BaLiCd_12_ compound compared to that of BaCd_11_. The crystal orbital Hamilton population curves (COHP) for the averaged selected interactions of **(B)** BaLiCd_12_ and **(D)** BaCd_11_. For BaLiCd_12_, the Ba–Cd interactions are underoptimized at the Fermi level, while the Cd–Li and Cd–Cd interactions are almost optimized at the Fermi level. In contrast, for BaCd_11_, all interactions are under-optimized at the Fermi level.

The atom-projected electronic density of states (DOS) for BaLiCd_12_ and BaCd_11_ are shown in Figures 2A,C, respectively. For BaLiCd_12_, a dip of the total DOS to ca. Three states eV^−1^ unit cell^−1^ can be observed at around *E*−*E*_*F*_ = 0.19 eV. In the rigid-band approximation, shifting the Fermi level ca. 0.2 eV would correspond to seven additional electrons per unit cell, or 0.88*e* ^−^ per formula unit. For BaCd_11_, a dip to ∼5 eV^−1^ unit cell^−1^ can be observed right near the Fermi level. However, neither are deep enough to be identified as pseudogaps.

In both compounds, the states in the vicinity of the dips are primarily contributed by the s-orbitals of Cd and Ba. Actually, the density of states plots of both compounds exhibit little structuring, with no gaps in the range of −3 eV *< E*−*E*_*F*_ *<* 9 eV. This is the characteristic of metallic bonding, indicating the absence of localized states or lone pairs. It appears that both systems are stabilized by the interaction between the delocalized electrons.

The crystal orbital Hamilton population curves (COHP) for selected averaged interactions in BaLiCd_12_ and BaCd_11_ are plotted in Figures 2B,D. For BaLi_2_Cd_11_, the Ba–Cd interactions are under-optimized at the Fermi level, while the Cd–Li and Cd–Cd interactions are almost optimized at the Fermi level. In contrast, for BaCd_11_, all interactions are under-optimized at the Fermi level. The optimized Cd–Li and Cd–Cd bondings in the cubic BaLiCd_12_ compared to the under-optimized bonding in the BaCd_11_ phase could be an indication of higher stability of the former. Given the similarity between the COHP curves of the Ba–Cd contacts in both compounds, the bond strength of Cd–Li and Cd–Cd in the icosahedron might be the driving force of the transition between these two phases. This inference is in line with the geometric explanation in the previous subsection.

## 4 Summary and outlook

The bonding characteristics of the Cd-rich phases, BaLi_2*.*1_Cd_10*.*9_ and BaCd_11_, both made from Cd flux, were studied both experimentally and computationally. In addition to providing insight into the relative stability of the tetragonal BaCd_11_ and cubic NaZn_13_ structures, this study reports the discovery of a new phase BaLi_2*.*1_Cd_10*.*9_, expanding the variety of compounds that crystallize in the NaZn_13_ structure type. Combined with the first-principles calculations of the electronic structure of both phases, we found that the bond strength of Cd–Li and Cd–Cd within the icosahedron might be one of the driving forces leading to the stabilization of the cubic NaZn_13_ type structure in this section of the Ba–Li–Cd phase diagram.

## Data Availability

The datasets presented in this study can be found in online repositories. The names of the repository/repositories and accession number(s) can be found below: The corresponding crystallographic information files (CIF) for BaLi_x_Cd_13–x_ (x ≈ 2) and BaCd_11_ have been deposited with CSD, and the data for this paper can be obtained free of charge *via* http://www.ccdc.cam.ac.uk/conts/retrieving.html (or from the CCDC, 12 Union Road, Cambridge CB2 1EZ, United Kingdom; Fax: +44-1223-336033; E-mail: deposit@ccdc.cam.ac.uk). Depository numbers are 2184513 (BaLi_x_Cd_13–x_) and 2184514 (BaCd_11_).
